# Dietary assessment training: The Italian IV SCAI study on 10–74 year-old individuals’ food consumption

**DOI:** 10.3389/fnut.2022.954939

**Published:** 2022-08-17

**Authors:** Cinzia Le Donne, Raffaela Piccinelli, Stefania Sette, Deborah Martone, Giovina Catasta, Laura Censi, Francisco Javier Comendador Azcarraga, Laura D’Addezio, Marika Ferrari, Lorenza Mistura, Antonella Pettinelli, Anna Saba, Donatella Barbina, Debora Guerrera, Pietro Carbone, Alfonso Mazzaccara, Aida Turrini

**Affiliations:** ^1^CREA Research Centre for Food and Nutrition, Rome, Italy; ^2^National Institute of Health, Rome, Italy; ^3^Independent Researcher (Former Council for Agricultural Research and Economics (CREA)), Scansano, Italy

**Keywords:** dietary assessment method, training methods, e-learning, hybrid learning methods, professional community, innovative process

## Abstract

Dietary surveys are conducted to examine the population’s dietary patterns that require a complex system of databases, and rules for constructing the data matrix (precision, coding, deriving new variables, e.g., body mass index from individual’s height and weight, classes, e.g., age-class, socio-economic status, physical activity, etc.). Management of the data collection requires specialized fieldworkers to allow for the collection of harmonized and standardized data. In this way, only statistical variability is envisaged and any eventual biases are due to probabilistic distribution but data are not affected by inaccuracy. Training the fieldworkers is a crucial part of each dietary survey. The idea to provide constant training throughout the whole survey period, from the preparatory phase to the data collection phase, relies on the necessity to train fieldworkers and monitor the skills acquired during the study, in addition to helping fieldworkers to gain the necessary experience. This study aims to relate the experience in conducting the course path to high specialized interviewers who carried out the cycle devoted to the 10–74 age class of the fourth nationwide food consumption study in Italy (IV SCAI ADULT) according to the European Food Safety Authority (EFSA) guide. A course path was structured in three steps corresponding to the preparation, pilot, and collection phases. The whole path achieved the goal of collecting data related to 12 individuals by each participant, with an overall success rate (successful trainees/total participants) of 16.8% (84 out of an initial 500). The study aimed to provide good quality data in the short term and a highly specialized community in the long term. Surveillance nutritional systems can count on a highly skilled community, so decision-making in public health nutrition and a sustainable and healthy food system can rely on this infrastructure.

## Introduction

Eating patterns in a population can be estimated through dietary surveys in which open-ended assessment methods such as diaries and interviews, or semi-quantitative food frequency questionnaires are included (1). A harmonized dietary survey methodology, combined with a standardized operational procedure for conducting the study, is crucial to ensure the comparability of the results with those of other national surveys and the accuracy of the information, thereby reducing uncertainty and thus increasing the reliability of the results (1).

Dietary assessment deals with a complex system composed of several elements: underpinning databases, codification rules, software, and management capacity, all of which need specialization and standardization of fieldworkers as reported in the scientific report supportive of the EU-Menu program (1). This has also been acknowledged in the literature related to nutritional epidemiology (2, 3) and several experiences in clinical trials and, in general, studies where diet and health are investigated (4). Understanding the dietary patterns (i) includes several target variables (foods, energy, and nutrients, other food components), and (ii) requires several explanatory variables (age, gender, anthropometric measurements, socio-cultural and economic characteristics, lifestyle, preferences, attitudes, beliefs, organization of household food-related activities, etc.). Moreover, dietary patterns influence several domains: imbalanced diets, acute and chronic exposure to substances that affect health, dietary-related non-communicable diseases, as well as sanitary expenditure, etc. Furthermore, food consumption has impacts on the rest of the food system elements such as production, processing, distribution, and other basic activities related to food marketing and access; food waste management including that of waste generated by food-related activities of households (e.g., packaging disposal), have consequences on the “health of the planet” which in turn can have effects on human health (4).

It is known that complex population studies require mastery to manage the several components mentioned above. Furthermore, the standardization of procedural approaches in order to produce comparable data is of the utmost importance considering that results are derived from cross-comparison of different groups (5).

Harmonization and standardization of dietary measurement methods and procedures in such a complex context require an *ad hoc* structured information system made up of databases (food nomenclatures, portion sizes, food atlas, recipes) and methodological tools (quantification methods, food coding systems, nutritional status assessment), as well as data processing to extrapolate what to evaluate as validated dietary data according to the defined rules) (1).

The first experience in undertaking a multi-phase training course covering the whole food consumption study of individuals in the age group of 3 months to 9-year-old was carried out from 2016 to 2019 (5). No other examples of this kind of experience are available in any other NCBI (National Center for Biotechnology Information) databases.^1^

In this second case study as well, the idea is to establish a community of professionals specialized in dietary data management in the nationwide dietary survey so that the implementation of a nutritional surveillance system can make use of a training/re-training system in the future (5).

**FIGURE 1:**
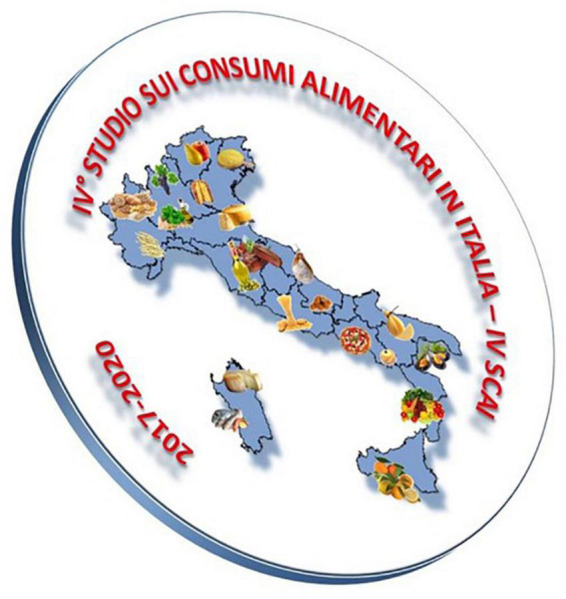
IV Study on food consumption in Italy (IV SCAI).

In this context, we propose this publication on the IV SCAI ADULT (Italian national food consumption survey on adult population from 10 up to 74 years-old; Figure 1) training course path. It follows the approach adopted in the survey involving 3 month to 9 year-old children (5) in order to share lessons learned, solutions applied to comply with the specific objective of the study (1), and future perspectives in this field.

Analogous to IV SCAI CHILD (5), this training course path aimed to build a community of highly specialized fieldworkers to conduct a survey on food consumption for the 10–74 age group according to the European Food Safety Authority (EFSA) guidelines (1).

## Materials and methods

CREA Research Centre for Food and Nutrition, the Ministry of Health, and the National Institute of Health planned a course path in order to provide theoretical and practical knowledge to collect food consumption data in the 10–74 age group at the national level. It was developed following the EU-Menu methodology (1) for carrying out a food survey according to the rules established at the European level.

The concept followed the previous course (5), but this time the application was different as the population group was aged 10–74, and the proposed methodology according to the EU-Menu program (1) was modified accordingly.

The main difference between these two paths was related to the measurement method (1). In fact, for the 10–74 age group, two repeated 24-h recall interviews (1) replaced two repeated food records on a paper diary (5), and associated questionnaires were adapted accordingly. Particularly, the background questionnaire was modified to include variables related to education, occupation, eating habits, and lifestyle. The International Physical Activity Questionnaire (IPAQ) (short version) was administered in this second study to subjects aged between 15 and 69 years. The instructions for anthropometric measures were adapted to the age class (6). Then, a Food Propensity Questionnaire (FPQ) was administered in only one version for the whole 10–74 age range (1). For 18 + adult subjects, questions about consumers’ attitudes were also investigated.

**FIGURE 2:**
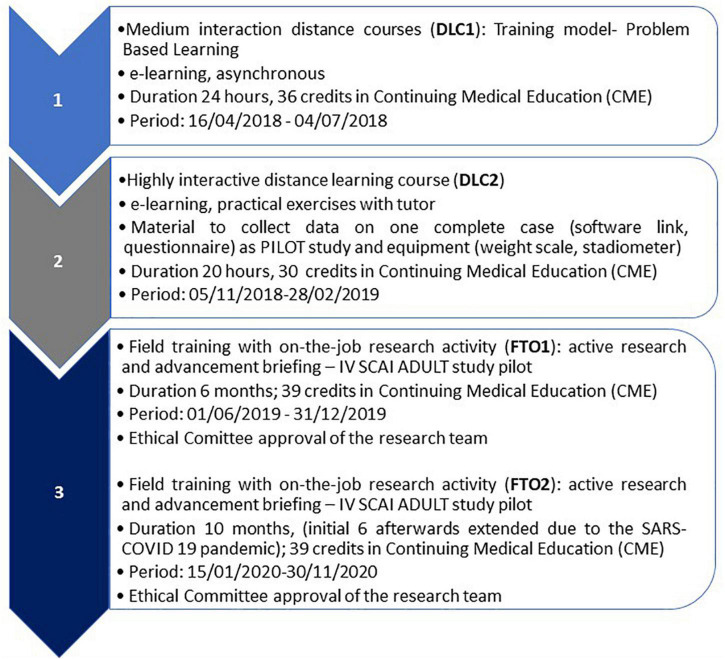
IV SCAI ADULT course path flow.

Thus, a hybrid approach was adopted to guarantee a solid experiential approach after the theoretical training, i.e., medium interaction distance learning (DLC1), highly interactive distance learning (DLC2), and field training with an on-the-job research activity articulated in two parts (FTO1 and FTO2). The whole course path is represented in Figure 2.

Medical doctors, biologists, dietitians, and nutritionists have been engaged through scientific societies, the official register of medical doctors and chambers of biologists were also involved, on a voluntary basis, because of their specific competence.^2^ Scores were assigned to the application by candidates to attend the DLC1 according to geographical criteria (balancing of areas), experience in the specific sector of nutritional surveys, the use of diet assessment tools, and participation in national surveillance programs. Subsequent courses were attended by trainees who successfully completed the previous steps.

### Medium interaction distance learning course (distance learning course 1)

Just as with the IV SCAI CHILD survey, the adopted approach for the first course in the path (DLC1) was inspired by the principles of Problem-Based Learning (PBL) (7, 8), where individual participants were activated by defining their own learning objectives and understanding and solving a problem, within their professional context. Trainees were expected to learn how to deal with the application of the knowledge base acquired to properly carry out dietary surveys on a national scale (Table 1).

**TABLE 1:** The 7 Steps of Problem-Based Learning in the medium interaction distance learning course—DLC1.

Steps of problem-based learning	Function	How to be carried out
Steps 1–4: Analysis of the problem	Reframing the presented situation, identifying the focus of the problem and sharing prior Knowledge and experience in relation to the problem	• Access the “exercise” resource and view the problem slides • Do the exercise, where you have to Answer questions about your previous knowledge and experience on the points raised by the problem
Step 5: Identification of learning objectives	Self-assessment of individual training needs through the identification of one’s learning objectives in relation to the proposed problem and their sharing within the group to select priority ones	• Reflect on which of your Learning Goals are achieved by performing the exercise. We will ask you to answer questions to identify the learning objectives that you will need to achieve in order to hypothesize a solution to the problem and you can compare them with those identified by the expert
Step 6: Research and study of materials	Independently search for study materials to achieve one’s learning objectives	• Access the “supporting materials” resource and try searching for your own study materials, always keeping in mind the “keywords” we have provided and the objectives to be achieved. You can use the bibliographical and sitographical indications provided • Access the “reading materials” resource selected by the experts. These are the essential materials you will need to study to complete the course • Access the “Tutorials,” where experts present a summary of the topics covered in the course. Follow them carefully, they will help you gain an overview of the course
Step 7: Solution of the problem	Solution to the problem, answering the questions posed at the end of it	• Access the “problem solution” resource and view the slides containing a solution proposal from the experts

The general aim and objectives were (i) to provide theoretical and practical knowledge for the survey of food consumption in the 10–74-year age group, through the acquisition of specific skills on the use of food consumption survey instruments and techniques necessary to carry out an individual food survey; (ii) to provide knowledge on how to use individual food consumption data in a public health context.

The learning unit contained:

• An introduction presenting the content of the unit.
• Specific learning objectives.
• A problem, based on a realistic scenario in relation to the topics of the course, is designed to be useful for the activation of the learning process and the application of the acquired knowledge.
• Supporting material, which the participant could use for further study and research, containing bibliographical references and web addresses related to the topic dealt with.
• Reading material (scientific articles, technical-scientific reports, legislative references, etc.) aimed at providing useful tools for solving the proposed problem.
• Video tutorials, represent the synthesis of the main discussion and study elements of the course, including films on how to perform the 24 h computer-assisted recall interview (multi-pass technique), and how to take anthropometric measurements according to the international guidelines (6).
• Practical exercise in the use of the data-entry software (FOODSOFT 1.0).
• Practical experience in conducting an interview. For this exercise, the participant was contacted by his or her tutor according to a booking schedule drawn up at the beginning of the course. The participant interviewed the tutor on the previous day’s food consumption (24 h recall method), applying the method acquired during the theoretical phase of the course.

The training course consisted of a series of preparatory activities that allowed access to the subsequent ones only if they were completed correctly (Table 2). Participants’ tutors monitored the activities *via* the online platform, enabling the release of the subsequent activities after quality control.

**TABLE 2:** Detailed description of activities, completion criteria and purpose of medium interaction distance learning course—DLC1.

Activities	Description	Completion criteria	Purpose
Entrance test	8 questions Answers multiple-choice	Answering questions 1 attempt only Running time: 15 min	Assessing one’s level of “knowledge” at the outset
Training exercise	Slides in Scorm format	View all the slides Answer all the questions Not timed	Analyzing the problem and activating prior knowledge and experience on the topic Self-assess one’s training needs through the identification of one’s learning objectives
Supporting materials	Sitography Bibliography Keywords	Viewing resources	Independently search for study materials to achieve their learning objectives
Reading materials	PDF documents selected and prepared by the experts/tutors	Viewing and studying materials	Acquire a basic knowledge of the topics covered in the course Hypothesize a solution to the problem
Tutorial 1-3-4 Tutorial 2	Slides with voice comments Scorm format slides with video	Viewing all slides of each tutorial Not timed	Access the summaries presented by the experts/tutors on the topics covered in the Course
First exercise	Delivery	Enter code generated after entering interview data	Practicing the interview with the 24 h Recall technique
Final exercise	Delivery	Enter code generated after entering interview data	Practicing the interview with the 24 h Recall technique
Solution of the problem	Slides with voice comments	Viewing all solution slides Not timed	Compare the solution hypothesis with the one presented by the experts/tutors
Self-evaluation post-test	8 questions Multiple-choice answers	Answering questions 1 attempt only Running time: 15 min	Assess the level of “knowledge” at the final stage. Incorrect answers indicate specific learning objectives to be revised
Final certification test	108 questions Multiple-choice answers	Pass the test by answering at least 75% of the questions correctly 3 attempts (waiting 16 h between attempts) Maximum running time: 200 min	Certifying successful completion of the Course
Evaluation questionnaire	Closed and open questions	Fill in all questions	Identifying strengths and weaknesses of the course for the continuous improvement of the distance learning offering
Questionnaire for evaluation of perceived quality	Closed questions	Fill in all questions	Collect feedback from participants in line with Continuous Education in Medicine (ECM) regulations

*There was also a “course evaluation questionnaire” and a “perceived quality assessment questionnaire,” the completion of which, together with the passing of the final evaluation test, allowed Continuous Education in Medicine (ECM) credits to be obtained.*

The estimated time for doing all the training activities and completing the entire course was 24 h, but it was not necessary to spend all that time online. The distance learning platform could be accessed according to personal and professional needs at any time within the 24-h period.

### Distance learning course 1 assessment method

The certification test consisted of 108 questions with multiple-choice answers (4 options), each question had only one correct option and a pass mark was established at 75%. A participant was given three attempts to get a pass mark. Participants were also required to have completed the training tests included in the course, which were:

– Entrance test (pre-test) to be completed at the beginning of the course, which allowed the participant to assess their prior knowledge of the topics to be covered in the course.
– Self-evaluation post-test of the level of knowledge acquired, taken at the end of the course. It was compulsory and allowed each participant to assess whether or not they needed to study the topics covered in greater depth.
– Practical exercise in the use of the software.
– Practical exercise of conducting the interview.

*There was also a “course evaluation questionnaire” and a “perceived quality evaluation questionnaire,” the completion of which, together with the passing of the final evaluation test, allowed Continuous Education in Medicine (ECM) credits to be obtained.*

### High interaction distance learning course (distance learning course 2)

**FIGURE 3:**
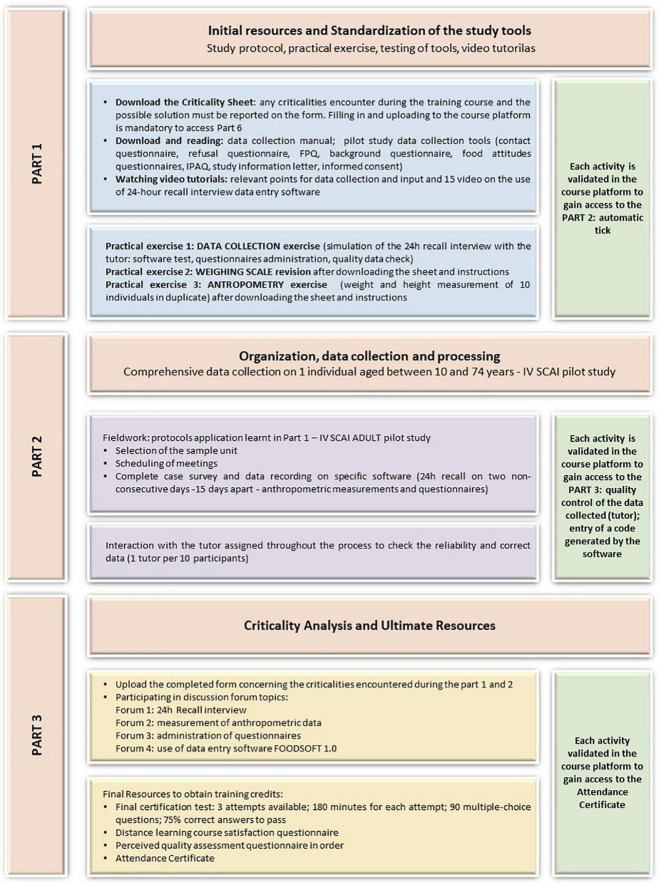
IV SCAI ADULT course path—Main steps of the highly interactive distance learning path—DLC2.

The high interaction distance learning course (DLC2) was devoted to the acquisition of skills to perform the pilot study of the Italian IV SCAI survey so as to test tools and techniques before starting the fieldwork (Figure 3).

The overall objective was to conduct the pilot study to collect individual food consumption data for the 10–74 age group (24 h recall method) according to the EFSA guidelines (1), organizing and carrying out data collection, handling the different stages from the preparation of instruments to data collection and assessing the quality of the data collected and defining corrective actions.

Each participant was supervised by a tutor who monitored the course progress on the online platform (1 tutor per 10 participants), urged compliance with the timetable for carrying out the activities, assessed the quality of the data collected, and decided if the participant was ready to continue with the next activity (if the judgment was positive) or needed to repeat the previous activity (in the case of a negative opinion).

The course was divided into three main parts, consisting of different activities which were made available in sequence and all propaedeutic in order to have access to the next parts.

#### Part 1. Initial resources and standardization of the study tools

It consisted of the preparatory phase for the conduct of the pilot study (part 2). The participant learned how to identify critical issues in advance and adopt corrective and timely solutions, which were agreed upon and modified for further cases. This phase included viewing and studying the material provided by the experts/tutors (handouts and videos) on the relevant points for food consumption data collection through the 24 h recall method (study protocol) and the use of data-entry software (FOODSOFT 1.0).

Furthermore, part 1 included three practical exercises, the purpose of which was to test in advance all the tools put in place for the execution of the national study:

– data collection exercise: conduct a telephone interview (approximately an hour-long) with the tutor on the previous day’s food consumption (24 h Recall method) and collect part of the questionnaires (food propensity questionnaire, background, and physical activity questionnaires) by entering the data directly on the software. All tutors were provided with a standardized interview format in order to assess adherence to the survey protocol and interviewer performance;
– weighing scales revision/calibration exercise: this activity involved the revision/calibration of the personal weighing scale (previously provided with the stadiometer) that the participant used to collect the weight of the subject selected for the pilot study (part 2), following the appropriate instructions in the section;
– anthropometry exercise: in order to ensure homogenous and comparable data, it is essential to use a standardized methodology and measurement instruments suitable for the purposes of the study. The aim of this exercise was to assess the replicability of the anthropometric measurements of the surveyors. To carry out this exercise, the participant recruited 10 persons between the ages of 10 and 74 (among friends, relatives, and colleagues) and collected weight and height measurements in duplicate. Each participant uploaded the appropriate format with the collected data onto the platform in order to obtain the tutor’s assessment and proceed with the course.

#### Part 2. Organization, data collection, and processing

This part involved performing the pilot phase of the IV SCAI Study—complete data collection on one individual aged between 10 and 74 applying the study protocol and extrapolating critical elements in the data collected. The activities to be performed were:

– contacting the individual, explaining the study, scheduling appointments.
– on the day of the meeting: signing the informed consent form, collecting anthropometric measurements, performing the 24-h recall interview (no more than 1 h) using the software, and administering the planned questionnaires.

After 15 days the procedure was repeated for the second 24 h recall.

Thanks to the online system designed for the IV SCAI study, all tutors performed quality control and corrections of the entered data in real time and in a standardized way. This allowed the immediate correction of possible deviations from the study protocol. The complete data approved by the tutors formed the sample for the pilot study.

#### Part 3. Criticality analysis and ultimate resources

This section of the course was dedicated to a discussion among all participants on the critical issues encountered throughout the course, with a focus on the practical experience of the pilot study. Four interactive thematic forums were set up: (1) 24-h recall interview; (2) measurement of anthropometric data; (3) administration of questionnaires; (4) use of the software. All remarks were analyzed by the tutors and the most relevant ones were taken into account in order to make changes in the final study protocol.

Each participant had to attend all the forums at least once to be able to access the ultimate resources. These were the course certification questionnaires: the final certification test (108 questions with multiple-choice answers), the course evaluation questionnaire, and the perceived quality evaluation questionnaire (as for the DLC1 course).

### Distance Learning course 2 assessment method

The evaluation method was the same as in the first course (DLC1): (i) completion of the final certification test (answering at least 75% of the questions correctly; 3 attempts; maximum running time: 200 min); (ii) completion course satisfaction questionnaire; (iii) and a perceived quality evaluation questionnaire, to obtain training credits for Continuous Education in Medicine (ECM).

The trainees did two 24 h recall interviews with an interval of 15 days; simultaneous recording (Computer Assisted Personal Interview) (9) using the dedicated software; administering questionnaires; measuring anthropometrics. Tutors attested trainees did all these tasks for successful completion of the course.

### Field training with on-the-job research activity (FTO1 and FTO2)

The last phase of this innovative training project consisted of a strong experiential component aimed at consolidating the theoretical knowledge acquired of the complex structure and implementing a nationwide seasonal population food consumption study.

The overall objective was (i) to conduct, as highly specialized surveyors, the IV SCAI Study—the fourth national study on food consumption in Italy in the 10–74 age group; (ii) to measure the weight and height of the subjects sampled; (iii) collecting, managing, controlling, and cleaning the data for the analysis of food consumption of the subjects sampled; (iv) estimate individual food consumption for the 10–74 age group.

The participants carried out the activities on the territory in two phases of the year (FTO 1 autumn-winter; FTO 2 spring-summer) using the FOODSOFT 1.0 (web-based data entry and processing software) managed centrally by the assigned tutor (1 tutor for every 10 interviewers).

### Field training on-the-job courses assessment methods

Participants passed each phase of the course if no fewer than 6 complete cases were entered. A positive or negative evaluation was carried out by each tutor who drew up an evaluation report based on the quality of material produced by each participant.

At the end of each phase, there was also a course evaluation questionnaire and a perceived quality evaluation questionnaire, the completion of which, allowed training credits (ECM) to be obtained.

The successful vs. candidate participants’ rate is a Key Performance Indicator (KPI). The “total number of collected data”/the “total number of trainees” ratio and “total number of collected data”/the “number of successful trainees” ratio were calculated as indicators of how difficult it was to achieve the sample size.

Moreover, the potential contribution of the community to further surveys was estimated.

Modifications were made to the course path vs. the IV SCAI CHILD one considering that a 24 h recall interview (a selective exercise for admission to the DLC2) was conducted using the appropriate software module. The trainee was given access to the software module *via* the web so that a meeting in person with the tutor, was not necessary. The 24 h recalls were checked through the system, for this reason, residential courses to attend in person were not planned.

Tutors were in charge of monitoring a group of trainees in all the phases (see the acknowledgment) in order to ensure the process of learning, harmonization, and standardization.

## Results

### Key performance indexes

The number of enrolled trainees by course is reported in Table 3. Those who completed the whole course path either participating in the FTO1 and/or FTO2 were highly motivated professionals and were 84 and 61 participants, respectively.

**TABLE 3:** Participation pattern described by enrolled, successful, trainees who accepted at different phases.

Course number	Module	Enrolled trainees	Successful/Invited to the next phase	Trainees who accepted to participate in the subsequent course	Success index	Withdraw rate
		A	B	C	(B/A)%	[1-(C/B)]%
1	DCL 1 high-interaction e-learning	500	312	193	62.4%	38.1%
2	DCL 2 high-interaction interactive e-learning	193	153	134	79.3%	12.4%
3	FTO 1 On-the-job training	134	84		62.7%	
	FTO 2 On-the-job training	86	61		70.9%	
	FTO 1 + 2 On-the-job training	134	84		62.7%	

DLC, Distance Learning Course; FTO, Field Training On-the-job.

Overall, the participation pattern described by enrolled, successful, and accepted trainees at different phases is reported in Table 3. It can be observed that the partial success index (successes/enrolled) on the whole course path was 12.4% varying, respectively, from the DLC1 success rate (312/500)% = 62.4%, withdrawal rate between DLC 1 and exercise was equal to [1-(193/312)]% = 38.1%, then DLC 2 had a success rate of (153/193)% = 79.3% and the withdrawal rate [1- (134/153)]% = 12.4%. Success rates slightly decreased compared to the DLC 2 in the two subsequent FTO 1 and 2 courses, reaching 62.7 and 70.9%, respectively. The overall success index was (84/500)% = 16.8%.

The evaluation of the course was all positive overall. Particularly, Table 4 shows how participants answered questions related to the structure and the usefulness of the FTO course. All three questions “How do you assess the relevance of the topics covered in relation to your updating needs?,” “How do you evaluate the educational quality of the ECM program?,” and “How do you evaluate the usefulness of this event for its training/updating?” gained at least 99% positive evaluations. It is interesting to note that the second FTO obtained higher evaluations than the first one, indicating an improvement of the quality with re-iteration of experience.

**TABLE 4:** Trainees’ evaluation of FTO1 and 2’ courses.

Question		FTO 1 %	FTO 2 %
“*How do you assess the relevance of the topics covered in relation to your updating needs?”*	Very relevant	47%	53%
	More than relevant	33%	33%
	Relevant	19%	14%
	Not very relevant	1%	–
	Not relevant	–	–
*How do you evaluate the educational quality of the ECM program?”*	Excellent	53%	76%
	Good	43%	20%
	Sufficient	4%	5%
	Partial	–	–
	Insufficient	–	–
*“How do you evaluate the usefulness of this event for its training/updating?*”	Very useful	46%	71%
	More than useful	33%	21%
	Useful	20%	8%
	Not very useful	1%	–
	Not useful	–	–

ECM, continuous education in medicine; FTO, Field Training On-the-job.

Considering the pilot study and the on-the-job courses 1,158 valid cases were collected. Therefore, the KPI related to the whole course path “total number of collected data”/the “total number of trainees” ratio resulted in (1,158/500) = 2, while the “total number of collected data”/the “number of successful trainees’ ratio was (1,005/84) = 12 (153 cases of pilot studies were not here included as those had 100% successful cases). This means that one case a month could be collected by one interviewer, which allows us to calculate how many fieldworkers would be necessary to achieve a given sample size.

## Discussion

Differences in implementing IV SCAI ADULT and IV SCAI CHILD course paths were mainly due to differences in methodology but also related to lessons learned. Particularly, residential lessons have been removed from the course path as organizing the meetings was quite difficult and the CAPI interview administration was considered to be more easily managed *via* the web.

The overall success rate was higher than the analogous results in the IV SCAI CHILD, i.e., 16.8% vs. 12.4% (5).

The IV SCAI ADULT course path to carry out individual food consumption surveys according to the EFSA’s EU-Menu methodology has contributed to the development of a specialized professional community covering infants to elderly age classes. The only age group excluded is the over 75 class (very elderly) but future developments are currently being discussed in the EFSA’s Network on Food Consumption Data.

Implementing the system on a wider scale to generalize the training method on the European level is not immediate as national contexts differ from each other in terms of geographical characteristics of the territory and regulation. These different aspects influence the sampling strategy used to represent the population, the sampling frames, and the rules to apply for complying with privacy statements.

Complementary questionnaires describing the sampled units and enabling the comparison of subgroups within the population must be adapted. What is more, the list of food differs in local products.

Local equipment can affect the methodology used, e.g., Paper And Pen Interview (PAPI) vs. Computer-Assisted Personal Interviews (CAPI) (9).

The idea to implement a surveillance program for public health purposes has a drawback as the World Health Organization (WHO) wrote: “the health workers are overburdened by excessive data and reporting demands from multiple and poorly coordinated subsystems” (10). This was an important reason for recruiting motivated fieldworkers to set up a stable community with internal rules to maintain the minimum number of subjects that minimize efforts to maximize the number of sample units to survey. The present study demonstrated that one case per month is feasible and it can be proposed to the healthcare system as an ordinary activity for skilled personnel to provide a national database with nutritional surveillance data.

One of the limitations encountered in the previous course was the complexity of the organization of 12 interactive frontal lessons that were held in the CREA Centres throughout the national territory (5). This part of the course involved a movement of people (tutors and participants) and tools for the execution of the meetings, causing an increase in costs both in terms of time and money. This previous experience led to the need to make a higher level of telematic interaction possible between tutors and participants, with the aim of reducing the time and costs of training a community spread throughout the country. The idea was to provide constant and interactive customized tutoring with: (i) realistic simulations of the application of the food consumption data survey method at different stages of the training course; (ii) access, through the platform, by the tutor assigned to monitor the progress of the proposed activities, so as to communicate any delays or give assistance in passing the compulsory activities; (iii) by organizing discussion forums at the end of each phase of the training course, which made it possible for participants to exchange information, compare common problems encountered, and apply standardized solutions to the system. This organization of the system made it possible to replace the face-to-face interaction between tutors and participants, shorten the time of the training course, and reduce the dropout rate.

Thanks to both training course paths it became possible to carry out the national study in Italy for the pediatric and adult population following the harmonized guidelines at the EU level. Moreover, the training courses even gave input to creating an international collaboration through a shared platform,^3^ which aims to strengthen the ability to replicate harmonized surveys by providing comparable and compatible data and, overall, to implement a continuous monitoring system in which new field interviewers are trained and previously qualified field workers can keep up to date.

The aim of extending the practice of undertaking a pan-European survey with the formation of a European community of specialized fieldworkers requires ensuring the training of new members and refreshing skills for all. It would be also useful if they could then in turn become trainers. In case, the training course path could be inserted into European Credit Transfer and Accumulation System (ECTS) (11).

Ultimately, the project experiments with a model that can generate an infrastructure for research activities in the field of food and nutrition surveillance, equipped with an information system for assessing the total quality of diets: nutritional adequacy, exposure to the risk of taking undesirable substances and environmental impact is essential. An open system for the introduction of future problems will also be required.

In such a context, it is expected that it will also be easier to experiment with new detection methods that can reduce the burden for detectors and participants, promoting an increase in the acceptance rate, while ensuring accuracy and precision.

This can lead to further beneficial effects, particularly the reduction of expenditures related to the economic cost of unhealthy diets [see e.g., (12)]. Literature reports examples of the need for training courses for the general public and stakeholders in the nutrition and food system field. As an example, see (13). This is in line with the healthy and sustainable diets issue widely treated in Willett et al. (14) publication. However, a community sharing interests, motivation, and expertise play a role in pushing toward a sustainable monitoring system that is expected to provide indicators for policymakers, local authorities, and other stakeholders (15).

## Future perspectives

The whole initiative IV SCAI is expected to save public funds mainly by embedding the survey in the public health system according to the present experience or the development of a specific infrastructure to be explored in the future in the context of the European Strategy Forum of Research Infrastructure (ESFRI), possibly in conjunction with the activities conducted in collaboration with the METROFOOD-RI (Infrastructure for promoting metrology in food and nutrition),^4^ which is currently concluding the preparation phase and entering in the implementation one.

For the sake of the development of a large community, we hypothesize the former evaluation (5) of saving money on public health actions is still valid, in particular when comparing the cost of the previous national survey (16).

Based on the innovative experience gained in organizing the distance training of interviewers for the Italian food consumption national study, two international projects have been launched.

The projects “Training in dietary assessment and sharing platforms for monitoring population food consumption habits in a long-term perspective (surveillance and continuous training) (TRAIN-DIE 2019–2020)” and “Sustainability of the platforms for monitoring population food consumption habits and pilot study on the web- and the computer-based 24-h dietary recall tools (SUP-DIE 2021–2023)” were proposed by CREA Research Centre for an e-Learning course, which was designed and completed to be available to many different users interested in collecting that complex set of data represented by individual dietary habits and related factors. The training of personnel to carry out these surveys, through a shared platform, will strengthen the ability to replicate harmonized surveys by providing comparable and compatible data and, overall, to implement a continuous monitoring system in which new field operators/interviewers are trained, while previously qualified interviewers can update their knowledge. The training system is designed to make future expansion possible, to understand the methodology in general, which could be expanded to new techniques or new tools and the experiences of individual countries, and to inspire creativity in the planning of studies that takes into account the specific characteristics of each reality.

At the moment, the e-learning course includes the description of the methodology and organization of the fieldwork according to the EFSA guidelines (1) and the national case studies that report application examples of the guidelines in Italy and Serbia. The platform’s website is https://train-die.crea.gov.it/?lang=en.

## Conclusion

An appropriate training course path can help to ensure good quality data collected in dietary surveys. The skilled community is now there, so the specialized fieldworkers are potential trainees of the future, and the structure of the course, opportunely modulated, will be available in other contexts (graduating courses, post-graduate courses, professional training) and other nutritional studies including dietary assessment (epidemiological studies, targeted nutritional studies, clinical trials, and so on). Creating and maintaining the dietary data managers community is challenging but feasible. In this context, the cooperation between the CREA Research Centre for Food and Nutrition and the Italian National Health Institute (ISS) promoted and supported by the Italian Ministry of Health may represent the best model that can ensure continuous training for the professional community carrying out dietary surveys. The information system is expected to be supportive of the decision-making process in public health nutrition and in turn a more sustainable food system would be developed as data surveillance systems generate data for health managers, food system actors, local authorities, and policymakers. In this way, the dietary assessment task can become more familiar and therefore easier to perform.

## Data availability statement

The original contributions presented in this study are included in the article, further inquiries can be directed to the corresponding author.

## Ethics statement

The studies involving human participants were reviewed and approved by the National Institute of Health Ethics Committee (Rome, Italy). Written informed consent to participate in this study was provided by the participants or participants’ legal guardian/next of kin.

## Author contributions

AT proposed and developed the concept together with GC, LC, FJCA, LD’A, MF, CLD, DM, LM, AP, RP, AS, SS, DB, DG, PC, AM, and AT first draft and all subsequent versions. GC, LC, FJCA, LD’A, MF, CLD, DM, LM, AP, RP, AS, SS, DB, DG, PC, AM, and AT revised and approved all versions. GC, FJCA, LD’A, MF, CLD, DM, LM, AP, RP, and SS developed the course concept, prepared all the material for the course’s path, tutored participants, and managed the survey in all the phases. LC developed and supported the part of the course devoted to anthropometric measurements. AS supported all the parts concerning the consumer’s science. AM coordinated and co-designed the concept of the e-learning course implemented and supported by DB, DG, and PC supported the implementation of the e-learning courses. All authors contributed to the article and approved the submitted version.

## Footnotes

1
https://www.ncbi.nlm.nih.gov/

2
https://www.frontiersin.org/articles/10.3389/fpubh.2021.590315/full

3
https://train-die.crea.gov.it/?lang=en

4
www.metrofood.eu

## CREA Research Centre for Food and Nutrition

Course project staff: Aida Turrini (scientific responsible; course director); Giovina Catasta; Laura Censi; Laura D’Addezio; Marika Ferrari; Cinzia Le Donne; Deborah Martone; Lorenza Mistura; Raffaela Piccinelli; Anna Saba; Stefania Sette.

Teachers/Tutors: Giovina Catasta; Laura Censi; Francisco Javier Comendador Azcarraga; Laura D’Addezio; Alessandra Durazzo; Marika Ferrari; Cinzia Le Donne; Deborah Martone; Lorenza Mistura; Raffaela Piccinelli; Laura Rossi; Anna Saba; Stefania Sette; Dario Berardi (Dev4U Srl).

Scientific Secretariat: Cinzia Le Donne; Raffaela Piccinelli; Stefania Sette.

Management Secretariat: Antonella Pettinelli.

### National Institute of Health

Course project staff: Donatella Barbina; Debora Guerrera; Alfonso Mazzaccara; Pietro Carbone;

Course Director: Umberto Agrimi.

Teachers: Francesco Cubadda; Marco Silano.

Scientific Secretariat: Francesco Cubadda; Marco Silano.

Management Secretariat: Emiliana Falcone; Valeria Patriarca.

### Ministry of Health

Course Director: Rossana Valentini.

Scientific Secretariat: Francesca Calvetti; Delia Forte; Roberto Copparoni.

The authors thank Dr. Gaetana Ferri (Ministry of Health) for her enthusiastic support, Dr. Elisabetta Lupotto (CREA Research Centre for Food and Nutrition) for her supportive commitment.

#### Participants

Our immense gratitude goes to all participants for their great and competent commitment in completing the IV SCAI 10–74 years old individuals: Nicole Acquaviva, Maria Albini, Rosaria Amabile, Enrico2 Apreda, Alice Baganha Sabatino, Cristina Baggio, Rossella Ballotta, Francesca Barberi, Sandra Bassini, Francesca Borghi, Giuseppa Bruno, Filomena Capasso, Vincenza Castiglia, Ketty Ceccarelli, Sara Ciacci, Lisa Colladet, Anna Maria Covarino, Marta D’Ambrosio, Ignazio Dei, Giulia Dellacostanza, Maria Assunta Di Cesare, Flora Di Tommaso, Emanuela Alessandra Donghi, Antonella Ferrigno, Paola Golzio, Marina Infante, Gianna Lia Innocenti, Domenica Iofrida, Valeria Laganà, Elisa Lazzarino, Carmela Legorano, Silvia Lisciani, Teresa Loiacono, Rosanna Macaluso, Manuela Maione, Monica Maj, Cecilia Mari, Valeria Marmo, Domenico Marotta, Piero Maruccia, Nicola Marulla, Marika Massaro, Guido Antonello Mattera Ricigliano, Maria Rita Milone, Antonio Molinaro, Mariantonia Monni, Laura Morisi, Patrizia Orsini, Giuseppa Pacella, Laura Parravicini, Franca Pasticci, Alessandro Pinto, Clorinda Pittalà, Angelica Pizzolante, Carolina Poli, Lucia Pomaro, Antonio Pratesi, Angela Pugliese, Sofia Pugnaloni, Marina Putzolu, Sara Quattrini, Valeria Rebonato, Paola Rossi, Gaia Rovai, Daniela Russo, Carmen Santangelo, Sara Santilli, Agata Rita Santonocito, Sabrina Scelfo, Egeria Scoditti, Angela Silvestri, Giuseppa Smeralda, Vincenzo Sofia, Silvia Soligon, Erminia Solomita, Ottavia Sorace, Angela Spadafranca, Paride Travaglini, Francesca Trinchella, Maria Trippa, Viviana Vecchio, Lorella Vicari, Sara Vignozzi, Laura Zangari.

The training course for the skill of highly specialized interviewers for the IV SCAI study was developed within the EU-Menu program of EFSA; An experiment of generalization of the Courses technique was carried out in the TRAIN-DIE and the SUP-DIE projects supported by the Central European Initiative (CEI).

## Abbreviations

DLC: Distance Learning Course
FTO: Field Training On-the-job
ECM: Educazione Continua in Medicina (Continuous Education in Medicine).
